# Homœopathy in Veterinary Practice

**Published:** 1896-11

**Authors:** Jacob Helmer

**Affiliations:** Scranton, Pa.


### HOMŒOPATHY IN VETERINARY PRACTICE. WHY I DO NOT PRACTISE IT.[1]

BY JACOB HELMER, D.V.S.,
SCRANTON, PA.

SYSTEMS of healing, like those of religion, attract their following by virtues, real or imaginary. Attracted by superior virtue, intelligent and honest men have forsaken one form of religious belief for another, or have abandoned a mode of healing which circumscribed their ability and skill. This is right. It shows a spirit of progression. Investigation is the mother of progress.

Articles tending to show the advantages of homœopathy over the regular treatment in medicine, and the reports of success with remedies applied according to this system, have appeared in our journals as evidence of a progressive tendency on the part of the writer. But the experiences reported have to the extent of their influence tended to array homœopathy against the regular practice; that this was not the motive is evident from the diffidence as well as the acknowledgment of limited experience in this mode of healing. Charlatans have, however, thus arrayed the two systems, and, by dint of assertion and persuasion, have crowded themselves upon the field of veterinary medicine with the object of selling their wares. Compilations of veterinary homœopathic materia medica have

[1] Read before the Pennsylvania State Veterinary Medical Association, Reading, Pa., October 6, 1896.

been issued ostensibly with the same purpose of advertising and selling their wares. Translations of foreign homœopathic veterinary works are offered. Under the laws of competition such things are natural. It is called business. But we are the representatives of science in our field. By means of recorded observation we build the substantial edifice of veterinary science. We would not remove a stone from this edifice if we could not replace it with a better one. The question is, Can we to any extent reconstruct this edifice on the principles of homœopathy? My note-book discloses that during a period of two years we treated diseases with the homœopathic and regular methods in order to form an idea of their comparative value. By comparison we see quickly the perfections and imperfections of the things compared.

History shows that the *regular* or *rational* school of medicine has been a development unhampered by any restricted tenets. Its foundation-stones are reason, experiment, and experience. Its *aim* has been *truth*. It accepts truth from any quarter. Recognizing disease as an entity, it has developed the sciences that include the phenomena of causes, viz., anatomy, physiology, biology, pathology, morbid anatomy, and bacteriology. Operating in sympathy with nature's laws, it seeks to remove causes. It does not claim to cure disease, but recognizes the healing power of nature. It uses every reasonable means to accomplish results. It stands ready to reject the ideas and methods of yesterday if it can substitute better ones to-day. It is progressive. It is not an exclusive system. It has not discovered any universal law of therapeutics. It does not claim to have found any law in nature either of similars or opposites according to which drugs exclusively influence the animal organism. It has witnessed the birth and death of exclusive systems. It needs not to go outside of itself, in direct disobedience to the principles of any founder, and appropriate the good of all in order to save its very life. It does not owe its success to credulity and superstition. It has been the most successful. It has not been driven by time and discovery to the humiliating spectacle of modifying and eliminating itself until scarcely anything remains but its flag.

On the threshold of experience with any method of treatment we must first learn its principles, conform to its rules, and accept the conditions it imposes upon us. The work announcing and elucidating the principles of homœopathy was published by its founder, Samuel Hahnemann, in 1810. He named it *The Organon of the Healing Art.*

On page 103 of the *Organon* he says: 1. *Similia similibus curantur* (likes are cured by likes) is the only therapeutic law; "that is to say, the only salutary treatment is that method according to which a disease is combated by a medicine capable of creating in the healthy body symptoms most similar to those of the disease." 2. The totality of the symptoms is the only guide to the physician in the administration of remedies; "that is to say, every drug before it may be properly employed in treating disease must first have been administered to a person in health, and the symptoms produced thereby recorded in order that their similarity or dissimilarity may be compared with those from which a patient may be suffering for whose relief a drug is sought to be administered." "All that the physician may regard as curable in disease consists entirely in the complaints of the patient and the morbid changes in his health perceptible to the senses." 3. The only true method enabling the physician to select the proper remedies in disease is to prove them upon a person in health.

To be consistent with these principles and to obey this therapeutic law we must experiment upon the various classes of the lower animals to find what drugs will produce symptoms similar to the symptoms of disease we desire to cure. But has this been done for veterinary homœopathy (if I may be permitted to use the expression)? Some authors on veterinary materia medica are silent on this point. Another speaks of careful provings having been made, but we have seen no record. But we know that animals have been utilized by the regular school as well as the homœopathic to demonstrate the toxic power of remedies to be used in the treatment of human diseases.

But admitting for the sake of argument that careful provings have been made upon different species of the lower animals, the results obtained are meagre, since only the objective symptoms produced by the drug can be noted. But some of the objective symptoms thus noted are unreliable, especially such as relate to the disposition and movement of the animal. Here it requires the discrimination of the prover to determine whether this class of objective phenomena are the primary result of the drug or are secondary to some influence produced by the drug.

Hence the symptoms which may be considered reliable, obtained by proving drugs upon the lower animals, are those indicated by the pulse, pupil, conjunctiva, the visible mucous membranes, the skin, and the excrements of the body. But the therapeutic laws of Hahnemann demand all possible symptoms obtainable from the action of a drug on every organ and tissue of the body. The subjective symptoms are therefore indispensable in a system in which symptoms are everything and morbid conditions and causes are nothing. "They fancied," says Hahnemann, "they could find the cause of disease, but they did not find it because it is unrecognizable and not to be found, since by far the greater number of diseases are of a dynamic (spirit like) origin and nature; their cause therefore remaining unrecognizable."

Observe that in the second part of the law quoted from the *Organon* it declares that the totality of the symptoms is the only guide to the physician in the administration of remedies. To meet this ever-varying totality of symptoms, and to find their correspondences, it has been found necessary to conduct extensive provings. The *Homœopathic Encyclopædia of Pure Materia Medica* is an evidence. It consists of ten large octavo volumes of about 700 pages each; but if only the objective symptoms of the proven were necessary to establish the system of homœopathy, such extensive provings would not have been made. But the subjective symptoms have been considered indispensable both to better fulfil the law of *similia* and to find remedies that will do better work.

But limitation to the intelligent and successful practice of veterinary medicine may be shown in another way, and this directly attacks the integrity of the therapeutic law upon which homœopathy is based. In my experience the infinitesimal dose does not appreciably affect animals either in health or disease, but doses large enough to cause appreciable symptoms in the pulse, pupil, muscles, and mucous membranes, if administered in a disease characterized by the same or similar symptoms, will intensify those symptoms instead of relieving them. This you have observed in the use of belladonna, aconite, nux vomica, etc. Therefore the law of *similia similibus curantur*, practically followed as we must follow it in veterinary practice, becomes null and void.

A consideration of the breadth and scope of the rational system applied to the practice of veterinary medicine, when compared with the limitations naturally imposed upon the homœopathic system when it deals with the maladies of the lower animals, ought to weigh in favor of the former method, and, unless it can be shown that success is more common and certain in homœopathy, ought of itself alone to detract one from adopting a circumscribed method.

Homœopathy in veterinary practice, finally, means to become a merely routine practitioner. To rely for success upon a supposed specific, judgment, discrimination, and skill can only be exercised in the selection of the remedy. Symptoms are everything. Causes and morbid conditions are nothing. It is not necessary to know the nature of a remedy, how it acts, or upon what it acts. If its symptoms correspond to those of the disease, the physician has done his work well. This reduces the practice of medicine to a simplicity.

Homœopathic remedies consist practically of tinctures and triturates. The tinctures are about the same strength as those of the *U. S. P.* The metallic and insoluble remedies are prepared in milk-sugar and dispensed in this form. The strength of a remedy is its dilution or potency. To produce a uniform series of dilutions a scale was introduced by Hahnemann, called the centesimal scale. According to this scale, one part of the mother-tincture or powder is mixed with ninety-nine parts of alcohol or milk-sugar. This forms the first dilution or potency. One part of this dilution in ninety-nine parts of alcohol, the second dilution. The principle is that the first potency must contain one one-hundredth part of the strength of the remedy, and the succeeding potencies each one one-hundredth part of the preceding one.

But, more recently, another scale has been introduced—the decimal scale. By this scale one part of tincture is diluted with nine parts of alcohol and similarly with the powders, one part in nine of milk-sugar, to form the first potency. In respect to the size of the dose authorities vary; some recommend the higher potencies—those above the thirtieth; others the thirtieth potency; others the third dilution of tincture and the sixth of insoluble remedies. But an exception is made of carbolic acid, aconite, rhus tox, bryonia, and others which may be used in the first or even in the form of the mother-tincture.

In applying homœopathy with the object of testing its value in treating the diseases of the lower animals it was necessary to form at least two classes; these were the acute critical and the acute non-critical. Of acute non-critical cases one-half were treated with homœopathy and the remainder with no medicine. In non-critical cases of pneumonia treated with high or low dilutions, or with no medicine, I could find no substantial difference. The cases ran the course marked by various changes in each case according to the natural history of the disease and the age, temperament, and vitality of the patient. There were differences, but it was not reasonable to refer them to anything besides natural causes. There was no noticeable effect of the remedy upon the pulse, respiration, and temperature. Practically, in horses, I found no difference between the first or third or thirtieth potencies in non-critical cases of pneumonia. In acute non-critical cases of disease in the horse of whatsoever nature, the experience was the same. The hygiene and diet were made the best possible. The superiority of the regular treatment was established in its power to abort colds and incipient diseases of the respiratory tract. In some acute critical cases nature seems powerless when rational treatment may and does save life. Rational after-treatment lessens the convalescing period and prevents relapses. I had no success with homœopathy in cases of toxic poisoning; in cases where pain was an element to subdue; or in impaction of the bowels, anæmia, vitulary fever in cattle, azoturea, colic, chorea, diabetes insipidus, laminitis, tetanus, purpura, rheumatism, and collapse. I do not mean that cases so treated did not recover, but cases treated *placebo* recovered. But the action of the remedy could not be traced; no desired effect could be produced; my tools were not tools. Acute critical cases will do better without medicine, unless such treatment be intelligent and not a hindrance to nature. Regular treatment not intelligently used is worse than no treatment. Here homœopathy may win a few laurels, and somebody think they have made a valuable discovery. An unsuccessful regular practitioner in using drugs to alleviate disease can become a successful homœopathist. Nature's tendency to heal must be fully recognized. In cases in which the regular treatment was of no value in my hands, homœopathic treatment did not demonstrate its superiority.

In the study of the principles of homœopathy one is surprised at the large number of the same or similar symptoms produced by drugs of a dissimilar nature. Again, the number of diseases dissimilar in their nature and manifestation that are treated by the same remedy. Gunther mentions eighteen remedies useful in paralysis, and indicates that there are many more. Haycock uses arsenic in about fifty diseases of the lower animals; aconite in over thirty diseases. Do all these dissimilar diseases have a totality of symptoms to correspond to the totality of the symptoms produced in an animal by a single drug? But it occurred that the symptoms of a disease may be removed in an animal and the morbid condition remain. Again, drugs do not produce the same or similar symptoms in healthy animals that are produced by some maladies which are treated by these drugs. Strychnine does not produce symptoms like paralysis, but it is used in paralysis by homœopaths. If the therapeutic law of Hahnemann were true, most maladies would be incurable. Homœopathy is not based on nature, but upon nature's most superficial and variable phenomena—symptoms. No system can become scientific on such a basis.

A few years ago I joined a class in Christian science. In this system patients were cured of their maladies of whatsoever nature; ruptures were declared healed; tumors were dissipated; rheumatics threw away their crutches, and bed-ridden subjects walked again. It looked like an era of miracles revived. The founder of Christian science in America, Mrs. Mary B. G. Eddy, was a homœopathic physician. In her work, *Science and Health*, she says, speaking of fevers, such as scarlet, typhoid, etc., that the higher the dilutions given the better success. She says it is not medicine but mind that heals. From this experience Mrs. Eddy thought she had discovered another proof of the power of mind to heal. But Dr. Roberts Bartholow, in his work on *Materia Medica*, says, speaking of the same subject: "Since we have learned the natural history of certain fevers, such as scarlet and typhoid, we find they can be most successfully treated with little or no medicine." Here was the secret and the truth. Christian science condescended also to treat the lower animals. We tried it. It was "weighed in the balance and found wanting." Were it not for human ignorance and credulity Christian science would have no following. But side by side with the boasted cures of Christian science, I place the boasted cures of homœopathy, and refer both these systems to nature, the only healer. But what benefit can these systems hope to achieve in treating patients that can exercise neither faith nor imagination?

But the homœopathic physician of to-day is instructed in anatomy, physiology, pathology, and allied sciences. He tells you that under the privilege of expedients he may employ any means, a remedy or a dose, in order to effect a cure. But here the law of *similia similibus curantur* practically ends. Only in a very limited sphere is the great therapeutic law subservient, and in that sphere mind cure and homœopathy meet.

In order to correct a wrong impression, and one apparently shared by some physicians, let me state here that there is no allopathic school. Hahnemann coined the word allopath and applied it to those opposed to his principles. To be an allopathist would mean to be a member of an exclusive school. The principles underlying our school have been already mentioned. Again, we are spoken of as the "Old School," in contrast with the "New School," meaning homœopathy. If by this is meant that ours is a school of antiquated ideas and non-progressive, the name does not fit us. What valuable discoveries have been made in medicine that were not made by the regular school? No one will assert that homœopathy has placed a stone in the edifice of pathology or added any fact of value to medicine. The great therapeutic law of homœopathy was not original with Hahnemann. Paracelsus had announced it 300 years before Hahnemann had his practice and begun to experiment upon the action of drugs upon persons. The results obtained he called provings. To-day provings yet constitute the mass of homœopathic literature. By careful provings it is shown that drugs may produce almost an endless number of symptoms; from 2000 to 3000 having been recorded in some drugs. Dunham says it is scarcely possible to analyze the actions of belladonna upon the human system. But a comparison of these provings shows that many drugs of opposite nature will give the same proving. That the provings include the sensations of the organism that occur during the provings period (but independent of drug influence), there can be no doubt.

One thing, however, was original with Hahnemann—it was the idea of the infinitesimal dose; the potentizing of drugs. He believed that the higher the potency the greater would be the power of the drug. None were high potencies unless above the thirtieth. No known method of science can detect a trace of medicine in a potency above the fifteenth; scarcely above the third. It cannot be the medicine then that is so powerful to cure. It is now held to be the potentiality produced by giving the vehicle containing the drug a certain number of shakes. Twelve shakes for a tincture and so many turns for a triturate. The more shakes and turns the more power in the remedy, until it may become very dangerous. Every drug is held to have a certain definite molecular activity which by shaking is imparted to the vehicle. Thus the vehicle becomes the remedy. It in turn imparts its molecular activity to the diseased protoplasm, which it restores to a normal condition. But this modern and ingenious explanation has not and cannot be demonstrated. It is not, therefore, of any scientific interest. But as before mentioned, since the safety of the law of similars depends upon the infinitesimal dose, it becomes necessary to resort to ingenious theories to prove there is virtue in the medicine prescribed in this manner. But we are not informed why the vehicle does not impart its molecular activity to the drug, or why the drug as it grows infinitely less in higher dilutions yet imparts an increased molecular activity. Hahnemann taught that when an insoluble substance was raised the third potency in milk-sugar, to obtain the fourth potency alcohol might be used, since the drug at this potency becomes soluble. It is to be presumed that his sense of sight and taste gave him all his knowledge of chemistry.

There are upward of eight hundred remedies in the homœopathic pharmacopœia. The *United States Pharmacopœia* contains less than one-half that number. The remedies are drawn from every department of nature; some of them will be regarded with curiosity and surprise. Apis mellifiæ is made from the stingers of bees, lachesis from the venom of a poisonous snake, lyssin from the saliva of a mad dog, vulpis hepar from the liver of the fox, and mephites from the odor-substance of the skunk. Psorinum is prepared from the pus of the human itch. A tincture has been prepared from the pus of gonorrhœa and administered internally to cure that disease. The insect world furnishes the bedbug, cockroach, house-fly, head-louse, wood-louse, ant, potato-bug, spider, and others. These insects are crushed and their juices received into alcohol or milk-sugar. These tinctures are used for internal medication. The tendency to use the remedies and doses of the regular school of medicine has become general among progressive homœopathic physicians. A travelling man for a wholesale drug-house informed me recently of his having good customers in the homœopathic fraternity. That he sold more tablets to homœopathic than to regular physicians.

The homœopathic physician who a number of years ago urged me to practise veterinary homœopathy, and who treated me with homœopathic remedies, gave the following prescription to one of my acquaintances: Potassi iodide, ℥iv; stillingia fl. ext., ℥ss; syr. sarsaparill. co. ad., ℥iv. Sig., etc. I reproached him with, "Doctor, is this homœopathy?" His reply was, "What difference does it make, if the medicine will do the patient good?" These illustrations show that the law of *similia* is not sufficient and is only partly adhered to, and then in minor ailments of the human family and in maladies that require more nursing than medicine. The Homœopathic Society of New York, in 1879, by a vote of thirty-three to fifteen, resolved that in the treatment of disease, the formula *causa sublata tollitur effectus* (cause and effect) is often to be remembered and used to advantage (Browning on *Homœopathy*). In a recent number of the *Homœopathic Monthly* Dr. Duke makes the broad statement that the law of similars is not applicable to any diseases which are characterized by destruction of tissues, or where the cause cannot be removed, or to such as are due to chemical action, mechanical violence, or unhygienic surroundings (Browning on *Homœopathy*). The *Medical Investigator* (a homœopathic publication) in 1876 said reprovingly: "How many claiming to be homœopathists are entirely disregarding the law of *similia*. It is getting to be quite a rare thing to hear of a homœopathic practitioner conducting a serious case from beginning to end without using, as such, cathartics, sudorifics, diuretics, etc., in direct opposition to our law" (*Encyclopædia Brit.*, 9th ed.). Says a writer in the *Homœopathic Times:* "To give one or more persons a drug, and register all their peculiar fancies and ideas, does not furnish any reliable evidence of the real effects of the drug." Says one homœopathist: "The question of potencies seems to have aroused a spirit of contention in the homœopathic fraternity about as bitter as any between the old and the new." Dr. Kidd says: "I have cast aside dynamized drugs *in toto* as untrustworthy and unjust to the sick" (Browning on *Homœopathy*).

With the homœopathic house divided against itself, with modern medical discoveries tending away from and not toward the doctrine of "*Similia*," isopathy is in no scientific sense like homœopathy. With the achievement of excellent success in the field of veterinary medicine according to the rational principles of the regular school, with boundless scope for the exercise of inventive genius and investigation afforded by a system of medicine that is bound by no law, but is free to work out the secrets of nature in her department through the exercise of observation, experiment, and reason, and thus to eliminate error and arrive at truth, should we not be proud to be workers in such a field? To any who wish to investigate this matter I refer them to works from some of which I have quoted, viz.: *The Organon of the Healing Art*, *The American Homœopathic Pharmacopœia*, *The Homœopathic Encyclopædia of Pure Materia Medica*, Hahnemann on *Chronic Diseases*, Gunther, Haycock, and Boericke & Tafel on *Veterinary Materia Medica*, *The Encyclopædia Britannica*, 9th edition. Also, to an admirable *Essay on Homœopathy*, by Dr. Browning, of New York.

